# A Compilation of Drug Etiologies of Stevens-Johnson Syndrome and Toxic Epidermal Necrolysis

**DOI:** 10.7759/cureus.48728

**Published:** 2023-11-13

**Authors:** Isaac T Abulatan, Sage G Ben-David, Lery A Morales-Colon, Elisabeth Beason, Adegbenro O Fakoya

**Affiliations:** 1 Medicine, University of Medicine and Health Sciences, Basseterre, KNA; 2 Medicine, St. George's University School of Medicine, St. George's, GRD; 3 Cellular Biology and Anatomy, Louisiana State University Health Sciences Center, Shreveport, USA

**Keywords:** toxic epidermal necrolysis, stevens-johnson syndrome (sjs), stevens-johnson syndrome, sjs/ten overlap, etiologies, drugs, toxic epidermal necrolysis (ten)

## Abstract

Stevens-Johnson syndrome (SJS) and toxic epidermal necrolysis (TEN) are adverse reactions that affect the mucocutaneous surfaces by causing necrosis and detachment of the epidermis. The difference between SJS and TEN is in the percentage of the body surface area (BSA) affected. TEN is known to affect greater BSA than SJS. The pathogenesis of SJS/TEN is attributed to drug-specific cell-mediated cytotoxic reactions that directly and indirectly lead to keratinocyte apoptosis through mediators. Clinical presentation begins with influenza-like symptoms, with the disease affecting the skin, oral, ocular, and urogenital regions most frequently. Although SJS/TEN is mainly due to various drugs, infection and vaccination can also induce SJS/TEN. This review outlines a compilation of all drugs implicated in SJS/TEN cases based on studies, mainly in case reports and other study types. Drug classes implicated in SJS/TEN cases include antibiotics, anticonvulsants, antineoplastics, analgesics, and diuretics, among others. There is no fully established diagnostic modality for SJS/TEN; treatment is done mainly by withdrawing the offending agent. In addition to withdrawing the offending agent, a multidisciplinary care team is essential in managing these patients. Several pharmacologic modalities have also been proposed in treatment, but there is still insufficient evidence for the efficacy of one against the other.

## Introduction and background

Stevens-Johnson syndrome (SJS) and toxic epidermal necrolysis (TEN) are adverse reactions that affect the mucocutaneous surfaces, causing necrosis and detachment of the epidermis. SJS and TEN are known to be on a similar disease spectrum, with the difference being the amount of body surface area (BSA) affected. SJS affects less than 10% of the BSA, while TEN affects greater than 30% of the BSA [1]. It is classified as SJS/TEN overlap when 10-30% of BSA is affected [1]. In this review, we will use SJS/TEN to refer to the full spectrum of the adverse reactions to the disease.

The incidence of SJS/TEN varies, but it is extremely low. In the UK, the incidence of SJS/TEN was 5.76 cases per million per year [2]. In France, TEN incidences were 1.2-1.3 cases per million per year [3]. The incidence of SJS/TEN can be estimated to be between two to seven cases per million people per year [4]. Females tend to be more affected than males, with a female-to-male ratio of 2:1 [5]. There is also a higher incidence of SJS/TEN among patients with HIV and cancer. Incidences of SJS/TEN among HIV patients have been reported to be between 0.95 and one per 1000 individuals with HIV [6]. The increased incidence in HIV patients is suspected because of the multiple medications used, immune dysregulation, genetic polymorphisms, and infections. Mortality for SJS/TEN ranges from 10% to 50%, with more mortality associated with TEN than with SJS [7].

In addition to HIV and cancer, genetic predispositions also put one at risk for having SJS/TEN. Patients with human leukocyte antigen (HLA)-B*15:02 and HLA-B*15:11 have an increased risk of SJS/TEN with carbamazepine use, especially in Asian populations [8-9]. HLA-B*58:01 has been implicated with allopurinol-induced SJS/TEN in Asian and non-Asian populations [10]. Other HLA alleles that have been implicated with other drugs include HLA-A*31-01, HLA-A*24:02, and HLA-B*13:01 [11-13]. Genetic polymorphisms in the CYP2C19 gene coding for the cytochrome P450 isoform may also increase the risk of SJS/TEN with drugs like phenobarbital, phenytoin, or carbamazepine [14].

## Review

Pathogenesis

The pathogenesis of SJS/TEN has yet to be understood entirely. Research has shown that SJS/TEN is due to a cell-mediated cytotoxic reaction through a CD8+ cell that leads to keratinocyte apoptosis. These cell-mediated T-cell reactions are drug-specific and directed against the native form of the drug. It is proposed that the different drugs that cause SJS/TEN bind with the major histocompatibility complex (MHC) class I and T-cell receptors (TCR) and lead to the expansion of drug-specific cytotoxic T cells that cause keratinocyte apoptosis both directly and indirectly through the release of cell mediators [15].

As noted, keratinocyte apoptosis is caused by cytotoxic mediated reactions through drug-specific CD8+ cytotoxic cells and natural killer (NK) cells directly and indirectly through mediators. Some of the mediators that have been proposed for these reactions include Fas-ligand, tumor necrosis factor (TNF)-alpha, perforin/granzyme, TNF-related apoptosis-inducing ligand (TRAIL), and granulysin. Granulysin, primarily known to be produced by cytotoxic T-cells and NK cells, has been majorly implicated in SJS/TEN. In a study, granulysin was the most highly expressed cytotoxic molecule through gene expression in SJS/TEN patients. The level of granulysin in blister fluid has also been correlated with the severity of SJS/TEN. When granulysin was decreased, the cytotoxicity was also decreased [16]. Another mediator implicated in SJS/TEN is interleukin-15 (IL-15). IL-15 is known to increase in SJS/TEN and has been correlated with disease severity and hospital inpatient mortality [17].

Other studies have also pointed out necroptosis contributes to keratinocyte death in SJS/TEN. The necroptosis process occurred because of the annexin A1 produced by monocytes binding to formyl peptide receptor 1 (FRP-1), leading to SJS/TEN [18].

Clinical presentation

SJS/TEN often begins with a prodromal nonspecific symptom that looks like influenza. Symptoms include fever often greater than 38°C, malaise, odynophagia, photophobia, and conjunctival injection. Sometimes, skin involvement is the first manifestation of the disease, occurring with blistering or skin tenderness. Early stages of SJS/TEN cutaneous lesions begin with poorly defined erythematous macules with purpuric centers or diffuse erythema, which could be tender to touch. The lesions are often symmetric and spare the scalp, palm, and soles of the body. It often begins at the face and then spreads to the thorax. It often exhibits a positive Nikolsky sign (slippage of the top layer of the skin from the bottom layer when rubbed). As the disease progresses, vesicles and bullae form on the skin, which later slough off. The degree of skin involvement based on total body surface area (TBSA) is a marker of the disease's severity, so it should be assessed regularly. Mucosal involvement, generally oral, ocular, and urogenital, often occurs in about 90% of patients [19]. Ocular involvement often includes conjunctivitis with purulent discharge, corneal ulceration, anterior uveitis, panophthalmitis, and trichiasis. The acute phase of SJS/TEN lasts for eight to 12 days, affecting the mucous membrane and causing denuded skin. Re-epithelization begins after several days and takes two to four weeks to form [20].

Chronic progression of the disease leads to multiple organ damage. For the skin, it can result in hypo/hyperpigmentation, scarring, and chronic pruritus. Ocular involvement includes dry eyes, symblepharon, corneal scarring, and rarely blindness. Long-term urogenital complications include adhesion formation, urethral stenosis, scarring, and strictures. Bronchiectasis, obstructive disorders, and chronic bronchitis/bronchiolitis are common presentations for the pulmonary system. In addition, long-term psychiatric consequences such as anxiety, depression, and post-traumatic stress disorder have been reported [21].

Due to extensive skin detachment, acute complications include massive fluid loss, infections, electrolyte imbalance, hypovolemic shock with renal failure, pulmonary complications (pneumonia, interstitial pneumonitis), gastrointestinal complications, and disseminated intravascular complications (DIC).

Drug etiologies associated with SJS/TEN

The cause of SJS/TEN is most often due to drugs. It takes approximately four weeks of continuous medication use for SJS/TEN to develop. Additionally, infections like *Mycoplasma pneumoniae* and herpes simplex virus have been associated with SJS/TEN. Although rare, SJS/TEN has also been reported after vaccinations, especially in childhood [22]. In one-third of cases of SJS/TEN, the causes are idiopathic. This paper records a compilation of the drugs that have caused SJS/TEN. Table 1 below shows the classification of the medications that have caused SJS/TEN.

**Table 1:** Drugs reported to induce SJS/TEN. SJS: Stevens-Johnson syndrome; TEN: toxic epidermal necrolysis.

Drug classification	References
Antibiotics	[23-45]
Anticonvulsants	[46-55]
Benzodiazepines	[56-59]
Non-benzodiazepines	[60]
Sulfonylureas	[24,61,62]
Diuretics	[63-69]
Analgesics	[4,70-74]
Antidepressants	[75-80]
Xanthine oxidase inhibitors	[81]
Androgenic hormones	[82,83]
Antineoplastics	[84-99]
Immunosuppressants/immunomodulators	[100]
Corticosteroids	[101]
Antiparasitic drugs	[102,103]
Antiviral drugs	[104-110]
Antifungal drugs	[111]
Aggrenox	[112]
Antihistamines	[113]
Angiotensin-converting enzyme inhibitors & angiotensin receptor blockers	[114,115]
Aminosalicylates	[116]
Others	[35,117-121]

Antibiotics

Antibiotics are one of the classes of drugs that have been implicated in SJS/TEN. Among the antibiotics, sulfonamides are the ones that have been most implicated. The most common sulfonamide that causes SJS/TEN is trimethoprim-sulfamethoxazole (cotrimoxazole). Attached in Table 2 is a list of common antibiotics implicated in SJS/TEN.

**Table 2:** Antibiotics that have been associated with SJS/TEN cases. SJS: Stevens-Johnson syndrome; TEN: toxic epidermal necrolysis.

Antibiotic class	Antibiotics	References
Sulfonamides	Trimethoprim-sulfamethoxazole (cotrimoxazole) and sulfasalazine	[23-25]
Aminopenicillin	Amoxicillin/clavulanic acid, ampicillin, ampicillin-sulbactam	[26]
Fluoroquinolones	Ciprofloxacin, norfloxacin, and levofloxacin	[27-30]
Tetracyclines	Tetracycline, minocycline, doxycycline	[31-33]
Macrolides	Azithromycin, clarithromycin, erythromycin, roxithromycin, telithromycin	[34-37]
Cephalosporins	Cefepime, cefixime, cefuroxime, cefazolin and ceftriaxone	[35,38-40]
Other	Metronidazole, rifampin, clindamycin, vancomycin, nitrofurantoin	[41-45]

Anticonvulsants/Antiepileptics Drugs

Anticonvulsants have also been implicated majorly in causing SJS/TEN. Various alleles have been suspected to be associated with some antiepileptic drugs (AEDs) in causing SJS/TEN. Carbamazepine-induced SJS/TEN is associated with the HLA-B*1502 allele [46]. Oxcarbazepine was involved with HLA-B*1518 in another report on causing SJS/TEN [47]. In addition, another study implicated combinatorial AEDs that are more likely to cause SJS/TEN. Table 3 below illustrates some of the AEDs associated with SJS/TEN.

**Table 3:** Common anticonvulsants that have been implicated in SJS/TEN. SJS: Stevens-Johnson syndrome; TEN: toxic epidermal necrolysis; AEDs: antiepileptic drugs.

Classification	Anticonvulsants/AEDs	References
Older drugs	Phenytoin, valproic acid, carbamazepine, phenobarbital	[46,48]
Newer drugs	Lamotrigine, oxcarbazepine, zonisamide, rufinamide, clorazepate, eslicarbazepine, clonazepam, and levetiracetam	[47,49-54]
Combination drugs	Clobazam-gabapentin, phenytoin-gabapentin, valproic acid-gabapentin, clobazam-clonazepam, clobazam–valproic acid, fosphenytoin-lamotrigine, and lacosamide-levetiracetam	[55]

Benzodiazepines/Non-benzodiazepines

Benzodiazepines and non-benzodiazepines, which are used to treat many conditions, have also been implicated in SJS/TEN. Chlormezanone was discontinued worldwide in 1996 due to severe and rare side effects of TEN. Table 4 highlights the drugs in these categories implicated in SJS/TEN.

**Table 4:** Benzodiazepines/non-benzodiazepines implicated in SJS/TEN. SJS: Stevens-Johnson syndrome; TEN: toxic epidermal necrolysis.

Classification	Drugs	References
Benzodiazepines	Tetrazepam, chlordiazepoxide, clobazam, flurazepam	[56-59]
Non-benzodiazepines	Chlormezanone	[60]

Sulfonylureas

Some case reports have implicated glipizide and glimepiride in SJS/TEN [61,62]. However, European case-control studies have denied any significant risk of SJS/TEN with the use of sulfonylureas [24].

Diuretics

Table 5 lists some diuretic medications that have been implicated with SJS/TEN. Other case-control studies, however, do not believe there is a significant risk of SJS/TEN with diuretics [63,64].

**Table 5:** Diuretics that have been implicated in SJS/TEN. SJS: Stevens-Johnson syndrome; TEN: toxic epidermal necrolysis.

Classification	Drugs	References
Loop diuretics	Furosemide, torsemide	[65-67]
Thiazide	Metolazone	[63]
Potassium-sparing diuretics	Spironolactone	[64]
Carbonic-anhydrase inhibitors	Acetazolamide, methazolamide, dorzolamide, and brinzolamide	[68,69]

Analgesics

Some analgesics have been associated with SJS/TEN. This is often common with nonsteroidal anti-inflammatory drugs (NSAIDs). A study that examined a total of 1,868 reports of SJS adverse events associated with five NSAIDs showed that ibuprofen had the highest association with SJS. Ibuprofen also had the highest rate of hospitalization but the lowest fatality rate [70]. Table 6 below lists the NSAIDs implicated in SJS/TEN cases.

**Table 6:** NSAIDs implicated in SJS/TEN. SJS: Stevens-Johnson syndrome; TEN: toxic epidermal necrolysis; NSAIDs: nonsteroidal anti-inflammatory drugs; COX: cyclooxygenase.

NSAIDs classification	Drugs	Reference
Non-selective COX inhibitors	Diclofenac, ibuprofen, aspirin, piroxicam, naproxen, ketoprofen, tenoxicam	[71,72]
Preferential COX-2 inhibitors	Etodolac	[4]
Selective COX-2 inhibitors	Rofecoxib, celecoxib, etoricoxib,	[70]
Analgesic-antipyretic with poor anti-inflammatory action	Acetaminophen	[73,74]

Antidepressants

Antidepressants, which are commonly used to treat depression, have also been associated with inducing SJS/TEN. Table 7 lists some common antidepressants associated with SJS/TEN cases.

**Table 7:** Antidepressants that have been associated with SJS/TEN cases. SJS: Stevens-Johnson syndrome; TEN: toxic epidermal necrolysis.

Classification	Drugs	Reference
Selective serotonin reuptake inhibitors (SSRIs)	Sertraline, fluoxetine	[75-77]
Serotonin-norepinephrine reuptake inhibitors (SNRIs)	Duloxetine	[78,79]
Atypical antidepressants	Mirtazapine	[80]

Xanthine Oxidase Inhibitors

Allopurinol, a drug used in the first-line treatment of gout, is a common cause of SJS/TEN. A particular case of a 33-year-old male diagnosed with gout and subsequently started on allopurinol began to demonstrate symptoms of TEN after 10 days of use. The patient was also found to test positive for HLA-B*5801 [81]. It is important to note that this allele has been associated with SJS/TEN cases with allopurinol use.

Androgenic Hormones

Androgenic anabolic steroids: SJS was reported in the case of a 41-year-old patient abusing anabolic steroids. The patient had been participating in competitive bodybuilding for roughly 20 years while taking varying doses of anabolic androgenic steroids (AAS). Further, the case confirms that the patient was not on other medications besides AAS [82].

Danazol: Other androgenic drugs include danazol, which is like testosterone and can be used to treat endometriosis. Additionally, danazol has been approved as a second-line agent for treating hematologic disorders related to SLE, including thrombocytopenia. Danazol has been implicated in triggering SJS [83].

Antineoplastics Drugs

Many antineoplastic drugs have been associated with SJS/TEN cases, as shown in Table 8. SJS/TEN is also common when antineoplastics are combined. Among the combined antineoplastics that have been implicated are docetaxel/cyclophosphamide, tegafur/gimeracil/oteracil (TS-1), brentuximab vedotin, and enfortumab vedotin [84-86]. In some instances, combination therapy between ipilimumab (which targets CTLA-4) and a programmed cell death protein 1 (PD-1) inhibitor resulted in SJS/TEN [87,88].

**Table 8:** Antineoplastics that have been associated with SJS/TEN. SJS: Stevens-Johnson syndrome; TEN: toxic epidermal necrolysis.

Classification	Drugs	References
Taxanes	Paclitaxel, docetaxel	[89,90]
Hormonal agents	Tamoxifen, enzalutamide	[91-93]
Protein kinase inhibitors	Ribociclib, afatinib, erlotinib, gefitinib, vandetanib, imatinib, sorafenib, bortezomib, vemurafenib	[94,95]
Monoclonal antibodies	Cetuximab, panitumumab, rituximab, ipilimumab, pembrolizumab, atezolizumab, nivolumab	[94,96]
Alkylating agents	Treosulfan, chlorambucil, mechlorethamine, temozolomide, bendamustine, procarbazine	[97]
Topoisomerase inhibitors	Etoposide	[97]
Antibiotics, cytotoxic	Anthracycline, doxorubicin	[97]
Antimetabolites	Methotrexate, cytarabine, fludarabine, gemcitabine, capecitabine, cladribine-6, mercaptopurine, pralatrexate, pemetrexed	[97]
Biologic response modifiers	Aldesleukin, denileukin	[97]
Miscellaneous	Thalidomide, lenalidomide	[98,99]

Immunosuppressants/Immunomodulators

Mizoribine: This immunosuppressant is used to treat renal transplant patients, systemic lupus erythematosus (SLE), lupus nephritis, and several childhood nephrotic syndromes such as IgA nephropathy. It has been implicated in one case of SJS, which occurred in a 32-year-old Japanese woman after receiving mizoribine for treating SLE with lupus nephritis for six months [100].

Corticosteroids

Corticosteroids are a mainstay in treating patients with early-stage SJS/TEN. However, it is crucial to know that administering high-dose corticosteroids in certain patients may paradoxically induce SJS/TEN. Some of these patient conditions may act as predisposing factors, including collagen disease, autoimmune disorders, or brain tumors [101].

Antiparasitic Drugs

Albendazole: Albendazole is an antiparasitic drug that can treat hydatid disease and other infections caused by tapeworms. A case report details albendazole causing TEN in a 16-year-old female [102].

Ivermectin: An antiparasitic drug that is FDA-approved to treat a variety of internal nematode infections has been documented in several reports of SJS/TEN [103]. Therefore, its implication in this severe adverse effect is crucial to be aware of. It is also important to mention that although not FDA-authorized or approved, its use has rapidly increased recently as COVID-19 prevention and as a component of the COVID-19 treatment cocktail.

Antiviral Drugs

Antivirals have also been associated with SJS/TEN cases, as noted in Table 9. HIV patients are known to be at higher risk than the rest of the population from developing SJS/TEN.

**Table 9:** Antivirals that have been implicated with SJS/TEN cases. SJS: Stevens-Johnson syndrome; TEN: toxic epidermal necrolysis.

Classification	Drugs	References
Neuraminidase inhibitor	Oseltamivir	[104]
Nucleoside reverse transcriptase inhibitor (NRTI)	Adefovir and tenofovir	[105,106]
Non-nucleoside reverse transcriptase inhibitor (NNRTI)	Nevirapine & efavirenz	[107-109]
Synthetic nucleoside analog	Acyclovir	[110]

Antifungal

Terbinafine: In one case, terbinafine was known to induce SJS/TEN overlap. The likelihood of this association is weak but possible [111].

Aggrenox

Aggrenox is a combination drug of acetylsalicylic acid (aspirin) and dipyridamole, which works to inhibit platelet activation and is thus used as an anticoagulant often to reduce the risk of stroke in high-risk patients. At least one case of Aggrenox-induced SJS has been reported. One occurred in a 74-year-old Chinese woman who was switched from aspirin to Aggrenox after she had experienced a transient ischemic attack (TIA) despite treatment with aspirin. She developed acute-onset progressive SJS within 24 hours from the first dose [112].

Antihistamines

A case of SJS/TEN overlap induced by fexofenadine-pseudoephedrine (Telfast-D) was reported in a middle-aged woman on treatment for persistent allergic rhinitis and sinusitis. Fexofenadine, an antihistamine sold under the brand name Allegra, is an over-the-counter drug used to treat allergy symptoms. After two doses, she initially presented with a swollen lip, which progressed to diffuse blistering with a positive Nikolsky sign over the week. She was ultimately diagnosed with SJS/TEN [113].

Angiotensin-Converting Enzyme Inhibitors and Angiotensin Receptor Blockers

These drugs are rarely reported to be associated with causing SJS/TEN. However, one report mentions the onset of SJS in a patient after being recently prescribed ramipril for hypertension [114]. Another case implicated losartan, an angiotensin receptor blocker (ARB), in causing SJS in a geriatric patient with renal impairment [115].

Aminosalicylates

Mesalamine and sulfasalazine, which are known aminosalicylates, have been found to have a causal association with causing SJS/TEN. The estimated absolute risk of four cases per 100,000 users was found to develop SJS/TEN [116].

Other causes that have been implicated in SJS/TEN cases

Acetylcysteine

Acetylcysteine is an antioxidant and glutathione inducer that is utilized for the treatment of acetaminophen overdose. It has been implicated in a reported case of SJS [35].

Ursodeoxycholic Acid

A drug used in the treatment of primary biliary cholangitis, as well as various other cholestatic disorders, has also been rarely implicated in cases of SJS/TEN. One ursodeoxycholic acid (UDCA)-induced SJS/TEN case occurred in a 24-year-old female patient being treated for deranged liver function tests. However, it should be noted that she was also prescribed concomitant omeprazole and domperidone, which also could have contributed to the induction of the severe cutaneous reaction [117].

Strontium Ranelate

The anti-osteoporotic agent boasts an excellent safety profile, aside from several cases of inducing SJS/TEN. In one case, strontium ranelate triggered what was diagnosed as mild SJS with a good prognosis in a 70-year-old woman after one month of beginning drug use. Although classified as mild, she exhibited multiple itching erythematous macules and plaques, which progressed to involve the entire body with severe oral and ocular mucosa involvement. Overall, early diagnosis and treatment led to a good prognosis in this patient. Thus, this adverse effect should be considered with this drug use [118].

Other non-therapeutic agents/chemicals in the etiology of SJS include iodine contrast, iopentol, commercial cannabinoid oil, and carbamate [119-121].

Diagnosis and diagnostic markers

There are currently no established diagnostic criteria for SJS/TEN. Diagnosis begins with suspicion in those with fever, flu-like symptoms, or SJS/TEN skin-like changes recently exposed to any drug or offending agent that could have triggered it. Usually, it takes one to four weeks of drug use before developing SJS/TEN symptoms [122]. Skin biopsy could be done through a punch or deep shave biopsy to confirm the diagnosis and exclude other diagnoses. Early histopathologic changes will show apoptotic keratinocytes in the basal layer of the epidermis, along with a perivascular mononuclear inflammatory infiltrate composed primarily of T lymphocytes in the papillary dermis [4,123]. This infiltrate is not diagnostic and could be seen in other skin conditions. Direct immunofluorescence is always negative for SJS/TEN.

Assessment of the likely drug causality is vital in diagnosis, as early withdrawal of the offending agent is a good prognostic factor. Detailed history and clinical insight are required to pinpoint the offending agent that could have triggered the process. An algorithm of drug causality for epidermal necrolysis (ALDEN) has been created to assess drug causality, especially in those taking multiple drugs. The ALDEN score is based on six factors (time lag from initial drug intake to onset of reaction; the probability that the drug was present in the body at the onset of reaction; pre-challenge (administration of the same drug to the patient in the past)/rechallenge; dechallenge; type of drug (notoriety); other possible etiologic alternatives) [124]. The final score is from -12 to 10, and a score is assigned to each medication. The grading system notes ⩾6 as very probable, 4 to 5 as probable, 2 to 3 as possible, 0 to 1 as unlikely, and ⩽0 as unlikely.

Recent research is looking for diagnostic markers to aid in diagnosing SJS/TEN. Markers that have been considered in research include the rapid granulysin test, CCR7, RIP3, galectin-7, granzyme B, and interferon gamma (IFN-y) [125,126]. However, diagnostic markers have yet to be fully established as the main diagnosis marker. Differential diagnoses to consider and rule out when diagnosing SJS/TEN include erythema multiforme, acute generalized exanthematous pustulosis, phototoxic eruptions, and staphylococcal scalded skin syndrome.

Management

The first step in the management of SJS/TEN is dependent on the etiology of the disease. In infectious cases, treatment of the infection is essential. If drugs are the cause, withdrawal of the offending agent or drug is vital as prompt withdrawal decreases mortality [127]. As noted earlier, it takes about one to four weeks of continuous drug use for SJS/TEN to develop, so the exact temporal relationship of drug use must be considered when identifying the suspect drug. The ALDEN score is also an important tool that helps identify suspicious drugs, especially when multiple drug use occurs. Another crucial prognostic tool is the severity of illness score for TEN (SCORTEN). It is based on seven independent risk factors and is often used to predict mortality for SJS/TEN. The score should be analyzed within 24 hours of admission and on day three of the presentation [128].

After dealing with the primary source of the disease, the main treatment modality is supportive. A multidisciplinary care team is essential as SJS/TEN affects various organs. Regarding the skin, wound care of patients is vital in preventing further infection or complications. Wound care could be achieved through conservative means (the epidermis is left in place, and further non-adhesive dressing is used to help in healing) or surgical means (the affected epidermis is removed, and biologic membrane of xenografts, allograft, biosynthetic dressings may be added or not). No evidence exists for surgical management of wounds above conservative means [129]. Other supportive management is fluid replacement to prevent organ hypoperfusion, temperature management to prevent hypothermia, nutritional support through oral means or nasogastric tube if the oral membrane is affected, pain control, infection prevention, and ventilatory support in cases of acute hypoxic respiratory failure, and urogenital and ocular care.

Several pharmacologic modalities have been proposed to treat SJS/TEN. Some are systemic corticosteroids, intravenous immunoglobulin (IVIG), cyclosporine, plasmapheresis, and anti-TNF agents. Some evidence has shown both positive and negative plausibility to their use [125]. However, there is still insufficient evidence for the efficacy of one against the other.

## Conclusions

In conclusion, SJS/TEN are often adverse reactions that affect the skin and mucous membranes of the mouth, eyes, and urogenital region. The difference between SJS and TEN is in the percentage of BSA affected. The pathogenesis of SJS/TEN is due to a cell-mediated cytotoxic reaction that leads to keratinocyte apoptosis through direct and indirect mediators. A major mediator that has been implicated is granulysin. Drugs often cause SJS/TEN, but other etiologies like infections have been suggested. One-third of cases of SJS/TEN are also known to be from idiopathic causes. There is no established diagnostic modality regarding diagnosis, but apoptotic keratinocytes with inflammatory infiltrate are the key findings. Management of SJS/TEN is generally the removal of the precipitating agent and supportive treatment.
